# Role of PD-1 co-inhibitory pathway in HIV infection and potential therapeutic options

**DOI:** 10.1186/s12977-015-0144-x

**Published:** 2015-02-08

**Authors:** Vijayakumar Velu, Ravi Dyavar Shetty, Marie Larsson, Esaki M Shankar

**Affiliations:** Department of Microbiology and Immunology, Emory Vaccine Center, Emory University, Atlanta, GA USA; Yerkes National Primate Research Center, 954 gatewood Road, Atlanta, GA 30329 USA; Department of Pharmacology and Experimental Neuroscience, University of Nebraska Medical Center, Omaha, NE USA; Department of Clinical and Experimental Medicine, Division of Molecular Virology, Linkoping University, Linkoping, 58185 Sweden; Tropical Infectious Diseases Research and Education Center (TIDREC), University of Malaya, Lembah Pantai, Kuala Lumpur, 50603 Malaysia; Department of Medical Microbiology, Faculty of Medicine, University of Malaya, Lembah Pantai, Kuala Lumpur, 50603 Malaysia; Center of Excellence for Research in AIDS (CERiA), University of Malaya, Lembah Pantai, Kuala Lumpur, 50603 Malaysia

**Keywords:** HIV disease, PD-1 blockade, PD-1 pathway, SIV infection, T-cell dysfunction, B-cell dysfunction, PD-L1 and PD-L2

## Abstract

Virus-specific CD8^+^ T cells play an important role in controlling viral infections including human immunodeficiency virus (HIV) infection. However, during chronic HIV infection, virus-specific CD8^+^ T cells undergo functional exhaustion, lose effector functions and fail to control viral infection. HIV-specific CD8 T cells expressing high levels of co-inhibitory molecule programmed death-1 (PD-1) during the chronic infection and are characterized by lower proliferation, cytokine production, and cytotoxic abilities. Although, antiretroviral therapy has resulted in dramatic decline in HIV replication, there is no effective treatment currently available to eradicate viral reservoirs or restore virus-specific T or B-cell functions that may complement ART in order to eliminate the virus. In recent years, studies in mice and non-human primate models of HIV infection demonstrated the functional exhaustion of virus-specific T and B cells could be reversed by blockade of interaction between PD-1 and its cognate ligands (PD-L1 and PD-L2). In this review, we discuss recent advances in our understanding of PD-1 pathway in HIV/SIV infection and discuss the beneficial effects of PD-1 blockade during chronic HIV/SIV infection and its potential role as immunotherapy for HIV/AIDS.

## Introduction

T cells are activated upon ligation of T-cell receptor (TCR) with specific antigen peptide MHC molecule (pMHC) displayed on antigen presenting cells (APCs). Upon first signal, naïve T cells become activated and an additional co-stimulatory signal promotes T-cell differentiation and effector functions. TCR signaling in the absence of co-stimulation induces T-cell anergy and weak co-stimulatory signals or a preferential engagement of co-inhibitory pathways during T-cell priming *in vivo* can lead to T-cell tolerance [1-3]. Ultimately, the balance between the co-stimulatory and co-inhibitory signals shapes the fate of T-cell response. The co-stimulatory molecule CD28 and the co-inhibitory molecules cytotoxic T lymphocyte antigen-4 (CTLA-4; CD152) and programmed death 1 (PD-1; CD279) are particularly important for regulating T-cell responses [4]. Recently, the co-inhibitory molecule PD-1, gained much attention in viral immunology as it plays a significant role in establishment of virus-specific CD8^+^ T-cell exhaustion. PD-1 was identified as a gene up-regulated in a T-cell hybridoma undergoing apoptotic cell death, and was thus named programmed death 1 [5,6]. PD-1 is inductively expressed on CD4^+^, CD8^+^, NK T-cell subsets, B cells and monocytic cell types upon activation. In close similarity to other CD28 family members, PD-1 transduces a signal when engaged along with TCR ligation. The cytoplasmic domain of PD-1 receptor contains two tyrosine-signaling motifs, both of which may be phosphorylated upon receptor engagement. Phosphorylation of the second tyrosine, the immuno-receptor tyrosine–based switch motif, recruits the tyrosine phosphatase, SHP-2 and to a lesser extent SHP-1 to the PD-1 cytoplasmic domain [5]. Recruitment of these phosphatases leads to de-phosphorylation of TCR proximal signaling molecules including ZAP70, PKCθ, and CD3ζ, leading to attenuation of the TCR/CD28 signal [7]. PD-1 signaling prevents CD28-mediated activation of phosphatidylinositol 3-kinase, resulting in reduced Akt phosphorylation and glucose metabolism. The PD-1 ligands have distinct patterns of expression. PD-L1 (B7-H1; CD274) is broadly expressed on both professional and non-professional APCs, whereas PD-L2 (B7-DC; CD273) is expressed in a inducible manner only on dendritic cells (DCs) and macrophages [8]. PD-L1 is constitutively expressed on B cells, DCs, macrophages and T cells, and is upregulated upon activation. PD-L1 is also expressed on a wide variety of non-hematopoietic cell types, including vascular endothelial cells, kidney tubular epithelial cells, cardiac myocardium, pancreatic islet cells, glial cells in the brain, inflamed muscle, and keratinocytes and also immune privilege sites such as the placenta and eye [8]. Interferon α, β, and γ are powerful enhancers of PD-L1 expression on APCs, endothelial cells, and epithelial cells [8]. During pro-inflammatory immune responses, such as infection or transplant rejection, PD-L1 expression is intense and extensive [8]. PD-L1 expression is found in many solid tumors, and high expression is associated with poor disease prognosis [8]. Several recent studies suggested that PD-1–PD-L pathway plays an important role in exhaustion of anti-tumor as well as anti-viral CD8^+^ T cells during chronic infections [8-12].

Dysfunctional virus-specific T and B cell responses are the primary reason for the diminished immune control during chronic viral infections [13-15]. Chronic HIV/SIV infection is characterized by continuous viral replication in the majority of HIV infected individuals, which leads to disease progression but there are rare exceptions when individuals (elite controllers) can control virus in the absence of therapy [16]. Persistent Ag exposure impair immune functions in HIV/SIV and this is a feature shared with various other chronic infections, such as hepatitis C virus, hepatitis B virus, and certain cancers [17]. The prolonged antigen exposures during chronic infections give rise to T-cell exhaustion, which is characterized by loss of proliferative capacity and effector function [18]. Evidence show that pathogens successfully evade immunity by activating negative regulatory pathways that play an important role in maintaining peripheral tolerance and avoiding excessive immune activation under physiologic conditions. Complex mechanisms are involved in this T-cell dysfunction and PD-1 has been identified as a major regulator of T-cell exhaustion during chronic HIV/SIV infection. Blockade of the PD-1 pathway in non-human primate model of HIV infection can reinvigorate exhausted T cells, resulting in enhanced viral control during chronic SIV infection [11,19]. Notably, recent clinical studies have revealed that PD-1-directed immunotherapy is highly effective in cancer patients, demonstrating that PD-1 is a promising therapeutic target in humans [20]. In this article we review recent studies that examined the role of PD-1 pathway in immunodeficiency virus-specific T and B cell immune dysfunction and discuss the therapeutic benefit of blocking PD-1 pathway during chronic HIV/SIV infection.

## Review

### Role of PD-1 pathway during acute viral infection

PD-1 is induced on T cells upon TCR stimulation. The PD-1–PD-L pathway is central in the interaction between host defenses aimed at eradicating pathogenic microbes and microbial strategies that evolved to resist immune responses. During acute viral infection or vaccination, effective antiviral T cells acquire the ability to accomplish multiple effector functions (including cytokine production, cytotoxicity and proliferation) after antigen encounter. Eventually, the effector response contract into memory, which gain the ability to persist for a long time, and have the capacity to expand during secondary antigen exposure. During this time there is a lower density of PD-1 expressed on virus-specific CD8^+^ T cells, whereas during chronic viral infection the expression of PD-1 increases to higher levels and these T cells lose the ability to produce cytokines, lyse infected cells and proliferate in a progressive and hierarchical manner. In a mouse model of acute lymphocytic choriomeningitis virus (LCMV-Armstrong) infection, the virus-specific CD8^+^ T cells expressed lower levels of PD-1 as compared with the clone-13 LCMV infection, which establishes chronic viral infection [14]. In mice, during the first week of LCMV infection, PD-1 expression increased on activated virus-specific CD8^+^ T cells. If the infection is cleared, PD-1 expression quickly decreases and functional memory T cells are generated. In contrast, if the infection persist as with the LCMV clone13 strain, PD-1 expression remains high and T cell function decreases [14]. In addition, PD-L1 increases during chronic LCMV infection, with the most PD-L1 on virus-infected splenocytes. *In vivo* blockade of PD-1–PD-L1 interactions in chronically infected mice restores T cell function and enhances the viral control [14]. The involvement of PD-L1 in limiting immunopathology was clearly demonstrated when LCMV clone 13 infection gave a chronic LCMV infection in wild-type mice, whereas PD-L1-deficient mice died [14]. Those observations also apply to humans as several groups have demonstrated that PD-1 expression is higher on HIV-specific T cells, HBV-specific T cells and HCV-specific T cells [17] compared to T cells specific for nonpersisting viruses such as vaccinia [21,22] or influenza [23]. Blocking PD-1–PD-L interactions *in vitro* reverses the exhaustion of HIV- and HCV-specific CD8^+^ and CD4^+^ T cells and restores cytokine production and proliferation [21,24].

Notably, PD-1 expression by virus-specific CD8^+^ T cells is low in individuals with low HIV antigen levels such as elite controllers and similar pattern is found in resolved HCV and HBV infection [25,26]. The levels remain high in individuals that progress to chronic HCV or HBV infection [25,26]. Furthermore, the expression of PD-L1 and PD-L2 on hepatocytes is upregulated by HCV infection and by type 1 and type 2 interferons [25]. These observations from both animal and human studies indicate that PD-1 expression on virus-specific T cells may limit their antiviral functions and ability to proliferate [21,24]. In addition, T cells specific for other chronic viruses such as cytomegalovirus (CMV) and Epstein Barr virus (EBV), express moderate to high levels of PD-1, respectively [22,27-29]. This suggests that sustained viremia and high antigen load maintain high levels of PD-1 expression on CD8^+^ T cells. The factors influencing the expression of PD-1 other than high antigen levels are not yet fully understood. Consequently, it is important to determine if up-regulation of PD-1 and its ligands is a consequence of antiviral IFN response, a result from the T-cell activation and inflammatory cytokine production, or whether chronic antigens, i.e. HIV, HCV, HBV proteins, have the ability to directly up-regulate their expression.

### Role of PD-1 pathway during chronic HIV infection

The expression of PD-1 and the mechanisms involved in PD-1 regulation in recently activated versus exhausted T cells are still poorly understood. PD-1 is induced by the strength and duration of TCR-pMHC interaction and CD28 co-stimulation and sustained by chronic antigen stimulation. PD-1 and its ligands are also induced by gamma chain cytokines IL-2, IL-4, IL-7, IL-15, IL-21 [30] and TGF-β [31]. In a longitudinal study of HIV-infected subjects followed from the time of acute infection, PD-1 expression declined on CTL-specific for epitopes that had undergone mutational escape along with an increase in CTL polyfunctionality as measured by the capacity to produce multiple cytokines, whereas an increase in PD-1 expression and monofunctionality was observed over time for CTL directed against conserved epitopes [32]. These data indicate that repeated Ag-specific TCR stimulation plays an important role in modulating PD-1 expression in HIV infection. However, other mechanisms contribute to Ag-independent up-regulation of PD-1. The accessory HIV protein Nef was recently shown to up-regulate PD-1 through a p38 MAPK-dependent mechanism during infection *in vitro* [33]. Furthermore, data suggest that the expression of PD-1 and its cognate ligands is influenced by the cytokine microenvironment, which varies in different tissues and body compartments and likely play a significant role in PD-1 regulation *in vivo* [8]. TCR-independent up-regulation may significantly contribute to the higher PD-1 expression observed on bulk CD8^+^ T-cell population and correlate with markers of HIV disease progression [34]. A large percentage of HIV-specific CD8^+^ T cells express PD-1, and the expression of this receptor is elevated on a per cell basis during chronic HIV infection. A larger proportion of HIV-specific CD8^+^ T cells also express CD27 and CD45RO, indicating previous activation. PD-1 high CD8^+^ T cells lack the expression of the co-stimulatory receptor, CD28, effector functions such as perforin and granzyme-B secretion/killing, and express lower levels of CCR7 and CD127 (IL-7 receptor α), which are important molecules for the maintenance of memory T cells [35]. This phenotype suggests that the T cells are poorly functional, not transiting into memory cells, and particularly receptive to inhibitory signals rather than activation signaling. This fits with the CD8^+^ T cells in HIV infected individuals, which are dysfunctional with reduced proliferative capacity and effector functions [22,27,28]. Studies, showed that disease severity, as measured by viral load and declining CD4^+^ counts, correlated with both the level of PD-1 expression on HIV-specific CD8^+^ T cells and the percentage of cells expressing PD-1, thus providing a CD8^+^ T cell markers that correlates with disease severity [22,27,28]. The level of PD-1 expression was also associated with decreased CD8^+^ T-cell proliferation in response to *in vitro* stimulation with HIV antigen. Collectively, these data suggest that the level of PD-1 correlates with the extent of T-cell exhaustion. In addition, PD-1 expression on HIV-specific CD8^+^ T cells was reduced in patients undergoing effective highly active ART (HAART), consistent with the notion that high antigen levels drive PD-1 expression and functional exhaustion [36]. Until recently several studies including ours in non-human primates, documented the role of PD-1-PDL pathway in HIV/Simian immunodeficiency virus (SIV) infection. Importantly, studies in macaque model clearly demonstrated that blocking the PD-1–PD-L pathway during chronic SIV infection *in vivo* leads to functional restoration of virus-specific T and B cells. Collectively, these observations suggest that the PD-1–PD-L pathway plays a major role in functional exhaustion of virus-specific T cells during chronic HIV/SIV infection [11,37].

### Role of PD-1 pathway in HIV-specific CD8^+^ T cells

Cytotoxic CD8^+^ T cells represent the major component of adaptive immune responses against viruses. CD8^+^ T cells are heterogeneous population that are classified into different subsets that define the major steps in a process of memory T-cell differentiation and are classified as effector memory, central memory, or during chronic infections, exhausted T cells [38,39]. These multiple subsets display specific transcriptional programs and express distinct surface receptors and intracellular molecules, indicative of different requirements for stimulation, survival, homing potential, and effector functions [40,41]. HIV-specific CD8^+^ T cells play a major role in controlling HIV infection. Research findings [21,22,28] demonstrated that HIV and SIV-specific CD8^+^ T cells express high levels of PD-1 and that PD-1 expression correlated with HIV and SIV-specific CTL dysfunction, as CTL expressing high amounts of PD-1 had impaired proliferative responses to the cognate Ag *in vitro* [21,22,28,37,42]. In a group of untreated subjects and a group of SIV infected macaques, PD-1 expression correlated with viral load and disease progression [21,22,28,37,42]. Longitudinal analysis of HIV-infected subjects before and after initiation of antiviral therapy showed that control of viral load resulted in reduced PD-1 expression on HIV-specific CTL. The expression of PD-1 was shown to be higher in HIV progressors compared to slow progressors [21,22,28,37,42]. Non-human primate models examining the relationship between PD-1 expression and apoptosis showed that PD-1-expressing CD8^+^ T cells are more susceptible to both spontaneous and Fas-mediated apoptosis. Cross-linking of the PD-1 molecule with anti-PD-1 antibody preferentially triggered apoptosis in the CD8^+^ cells expressing high levels of PD-1 [29,43]. The PD-1 expressing HIV-specific CD8^+^ T cells had lower proliferative abilities and cells expressing very high levels of PD-1 were highly susceptible to death signals, suggesting that PD-1 expression leads to impaired survival in vivo [18]. In addition, there are indications that PD-1 expressing CD8^+^ T cells have defects in pathways associated with metabolic and mitochondrial function in CD8^+^ T cells during chronic HIV infection [27,43,44] and that they have shortened telomeric DNA and reduced telomerase activity [45].

**Figure 1:**
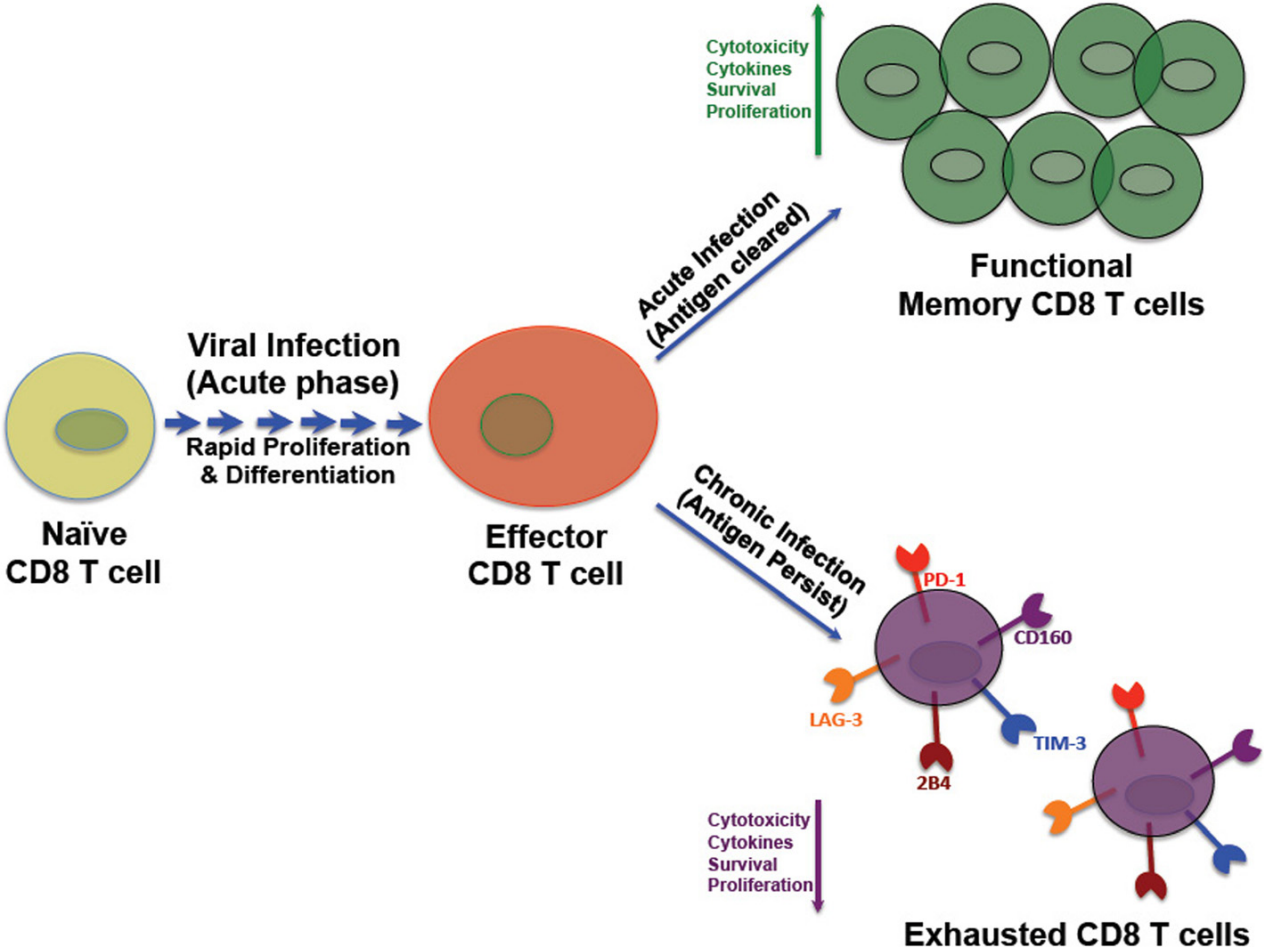
**Regulation of CD8** ^**+**^ **T cells after acute/chronic viral infection.** Highly complex network of co-inhibitory and co-stimulatory signaling pathways regulate the outcome of virus-specific CD8 T cells. High amount of antigen drives the exhaustion of virus-specific CD8^+^ T cells during chronic viral infection that is evidenced by the expression of multiple immune-inhibitory receptors. (Note: Exhausted T cells with loss of function may not control chronic viral infection). However, during acute viral infection the antigen is cleared from the host and the virus-specific CD8 T cells are fully functional and produce cytokines and have proliferative potential, survival and cytotoxicity to control viral infection.

Blockade of the PD-1 pathway by anti-PD-L1 antibody or by anti-PD-1 antibody resulted in enhanced HIV and SIV-specific CD8^+^ T-cell proliferation [21,22,28,37,42]. However, the restoration of HIV-specific CD8^+^ T-cell proliferation was relatively modest in some individuals indicating that only a minority of the HIV-specific PD-1-expressing CD8^+^ T cells could have their function critically inhibited by only PD-1, and are therefore effectively enhanced by a PD-1/PD-L1 blockade. One reason could be the expression of multiple inhibitory receptors on virus-specific CD8^+^ T cells (Figure 1), which make the functional restoration of these cells by PD-1 blockade alone impossible. A recent study support the fact that multiple inhibitory receptors are expressed (2B4, CD160, PD-1) on HIV-specific CD8^+^ T cells and the simultaneous blockade of both the receptors enhance the restoration of CD8^+^ T cells than blocking a single receptor alone [29,46]. A recent study in mouse clearly demonstrated that exhausted CD8^+^ T cells are co-regulated by complex pattern of multiple inhibitory receptors during chronic LCMV infection [47] and that cells expressing more than one inhibitory receptor might have affected the outcome of the PD-1 blockade. More studies are needed to demonstrate similar phenomenon in HIV infected humans and to better understand the regulation of PD-1 expression and function in activated and exhausted cells. Recently, NFATc1 was identified as an important factor in the regulation of PD-1 expression, thus providing a molecular mechanism responsible for the induction of PD-1 upon T cell stimulation [48]. Critical questions remain as to what distinguishes the regulation of PD-1 expression and function in exhausted compared to functional activated CD8^+^ T cells [49]. It will also be important to determine whether PD-1 expression is modulated by their ligands (PD-L1 and PD-L2), whose expression levels may vary over time during infection or in different tissues.

### Role of PD-1 pathway in HIV-specific CD4^+^ T cells

CD4^+^ helper T cells orchestrate both cellular and humoral arms of the immune response and may play a critical role in controlling HIV replication [50,51]. Chronic HIV infection is characterized by the rapid depletion of CD4^+^ T cells and defective HIV-specific CD4^+^ T-cell responses is the hallmark of HIV infection. Effective virus-specific CD4^+^ helper T cells are considered to be important in restricting viral replication during chronic HIV infection [52]. Defective CD4^+^ help leads to poor quality of CD8^+^ T-cell responses [51,52]. Recent studies suggest the role of PD-1 pathway in CD4^+^ T cell dysfunction similar to PD-1 pathway on CD8^+^ T cell [37,53,54]. PD-1 is also up-regulated on HIV-specific CD4^+^ T cells and its expression level correlated with plasma viremia and inversely with total CD4^+^ T cell counts [55]. In addition, the PD-1 levels were higher on HIV-specific CD4^+^ T cells compared with CMV-specific CD4^+^ T cells in the same subjects. Of note, the PD-1 levels on CD4^+^ T cells in HIV infected individuals were higher in lymph nodes than in peripheral blood [56]. There was also a strong correlation between PD-1 levels in CD4^+^ and CD8^+^ Gag-specific T cells demonstrating the involvement of PD-1 pathway in regulation of both CD4^+^ and CD8^+^ T cell types. Similar to PD-1, CTLA-4 another molecule of B7:CD28 family also mediates CD4^+^ dysfunction and are highly elevated on HIV-specific CD4^+^ T cells [53]. Importantly, recent data extended these observations by examining expression of PD-1, CTLA-4 and another regulatory receptor TIM-3, which has been shown to impair HIV-specific CTL responses [53,55]. Co-expression of PD-1, CTLA-4 and Tim-3 correlated more strongly with viral load compared to the expression of each receptor individually. These data collectively demonstrate that multiple inhibitory receptors co-regulate virus-specific CD4^+^ T-cell impairment [57]. Noteworthy, in contrast to HIV-specific CD8^+^ T cells, HIV-specific CD4^+^ T cells express CD28 and it has been demonstrated that PD-L1 blockade combined with CD28 stimulation had synergistic effect on enhancing HIV-specific CD4^+^ T-cell proliferation *in vitro* compared to targeting them individually. These demonstrate that the concurrent modulation of co-inhibitory and co-stimulatory receptors enhance the restoration of HIV-specific CD4^+^ T cell responses [58]. The transcriptional factor BATF is downstream of the PD-1 receptor signaling and play a key role in CD8^+^ T cell exhaustion and a recent study investigated primary HIV-specific T cells by silencing BATF expression and demonstrated that it augmented IL-2 production, suggesting that BATF may play an important role in impairment of CD4^+^ T cells in HIV infection [35,47]. Taken together, these data suggested that HIV-specific CD4^+^ T cells are controlled by complex pattern of negative regulation due to expression of multiple inhibitory receptors, and that the sets of inhibitory molecules controlling CD4^+^ and CD8^+^ T cell responses are only partially overlapping [57,59].

**Figure 2:**
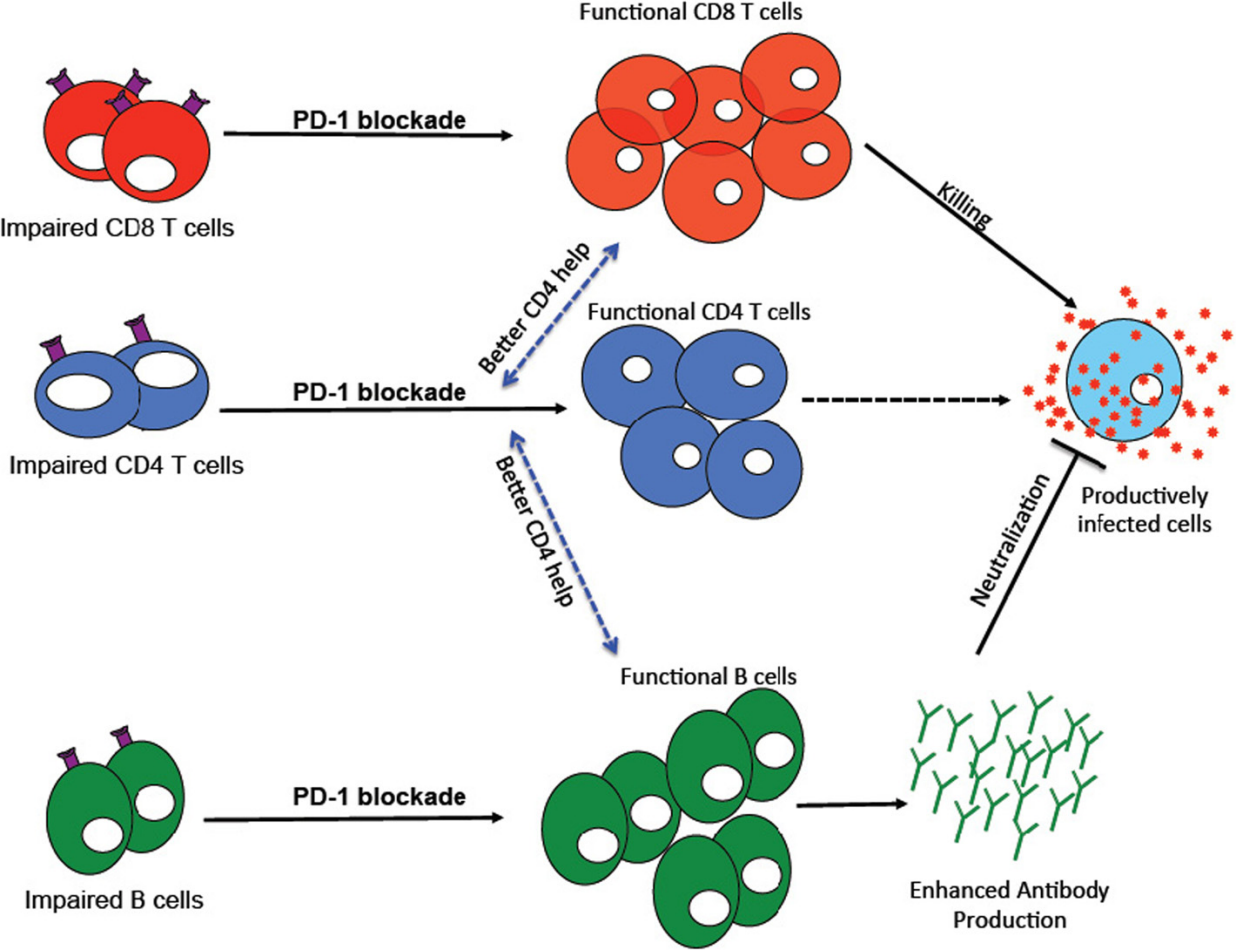
**PD-1 blockade enhances both T and B cell responses during chronic HIV/SIV infection.** PD-1 blockade mediated functional restoration of exhausted virus-specific CD8^+^ T cells gain their qualities to clear viral antigens and control chronic viral infection. Blockade of PD-1 in virus specific CD4^+^ T cells restore functions associated with CD8^+^ T cell help and B-cell activation. Impaired B cells restore functions and produce virus-specific antibody following in vivo PD-1 blockade.

Blockade of the PD-1 pathway with a PDL-1 blocking antibody increased HIV-specific CD4^+^ T-cell proliferation and similar effects were also seen in SIV infected macaques CD4^+^ T cells [53]. The CD4^+^ T-cell proliferative responses that follow blockade of the PD-1 pathway likely vary between individuals, with striking effects in some individuals that correlates well with consequent CTL responses [21,60]. Importantly, the blockade of PD-1 pathway with anti-PD-L1 antibody in *in vitro* cultures of CD4^+^ T cells activated with HIV antigens enhanced the secretion of several T helper cell lineage-specific cytokines (Figure 2), known to mediate numerous functions *in vivo* [58]. Potential role of PD-1 pathway in B-cell responses has been identified, as the PD-1 expression that is induced on newly activated CD4^+^ T cells is maintained and further increased in CD4^+^ T cells that interact with cognate B cells called T follicular helper cells (Tfh) [61-63]. Tfh cells express CXCR5 and migrate to the germinal center (GC) where they select high affinity B cells by providing help via IL-21 and CD40L, which help them to survive and differentiate into memory and plasma cells. Recent findings in HIV and SIV models suggest that PD-1^+++^ CD4^+^ T cells in the lymph node are similar to Tfh cells that interact with cognate B cells in the germinal centers of HIV infected humans and SIV-infected macaques [64-67]. These cells express very high levels of follicular markers (CXCR5, BCl-6, ICOS) including PD-1, which may interact with the ligands expressed on B cells in the germinal centers and required for effective B-cell responses [64,68-70]. HIV/SIV infection induces multiple changes in Tfh cell numbers and function, ultimately affecting the development of HIV/SIV-specific antibodies [71-74]. In addition, it has been reported that GC and memory B cells express PD-1 ligands PD-L1 and PD-L2 respectively Table 1 [73]. More importantly, PD-1 engagement suppresses follicular T-regs, which appear to express high levels of PD-1, and a subset of Foxp3^+^ cells that limit GC reactions [61]. Future studies are needed to delineate the regulation of Tfh cells, i.e. role of PD-1, which could inform vaccination strategies with the goal of eliciting broadly neutralizing antibody responses and to address the impact of PD-1 blockade on CD4^+^ T cells beyond antiviral treatment, which may further shape the quality of CD8^+^ and B-cell responses.

**Table 1:** **Summary of principal findings on the role of PD-1 pathway in HIV/SIV infection**

**PD-1 expression on SIV/HIV-specific T cells**	**Cell types**	**Reference**
Highly regulated on SIV/HIV-specific CD8^+^ T cells	CD8^+^ T cells	[21,22,28,37,42,75]
PD-1 expression on SIV/HIV-specific CD4^+^ T cells	CD4^+^ and CD8^+^ T cells	[21,37,53,56]
PD-1 is co-expressed with other co-inhibitory receptors	CD8^+^ and CD4^+^ T cells	[29,47,57,76]
PD-1 expression correlates with disease progression	CD8^+^ and CD4^+^ T cells	[21,22,37,60]
PD-1 expression on TFH cells	GC and CD4^+^ T cells	[65-68,70,71,73]
**PD-1/PD-L1 expression on B cells during chronic HIV/SIV infection**		
PD-1 is up-regulated on B cells during chronic SIV infection	Activated B cells	[10]
PD-1 is up-regulated on B cells during HIV infection	B cells	[77,78]
PD-L1 up-regulated in B cells	B cells	[79]
PD-L1 up-regulated in B regulatory cells	Bregs	[80]
High expression of PD-L1 on germinal center B cells	GC B cells	[73,81]
No change in PD-L2 expression on B cells	B cells	[73,81]
**PD-1/PD-L expression on innate immune cells during chronic HIV/SIV infection**		
PD-L1 expression on DCs	PDCs and MDCs	[82-84]
PD-L1 expression on monocytes	Monocytes	[79,82,85,86]
PD-L1 and PD-L2 expression on macrophages	Macrophages	[87]
PD-L1 expression on neutrophils	Neutrophils	[88]
**Effect PD-1 blockade during chronic HIV/SIV infection (both** ***in vitro*** **and** ***in vivo*** **indicated)**		
PD-1 blockade enhances proliferation of HIV-specific CD4^+^ and CD8^+^ T cells	CD4^+^ and CD8^+^ T cells	[21,22,37,42]
PD-1 blockade enhances cytokines by HIV- or SIV-specific CD8^+^ and CD4^+^ T cells	Cytokine^+^ CD4^+^ and CD8^+^ T cells	[11,58]
PD-1 blockade enhances SIV-ENV-specific antibody response	ENV-specific antibodies	[10,11]
PD-1 blockade in humanized mice model	CD4^+^ T cells	[54,89]
PD-1 PD-L1/L2 blockade enhances IgG production	GC B cells	[73]

### Role of PD-1 pathway in B-cell dysfunction during HIV infection

B cells play a key role in the adaptive immune response and are the source of the humoral immune response against a given pathogen [90]. Understanding their activation process is of major importance to develop effective vaccine-based strategies. B cells require at least two signals to engage their activation program in similar manner to T cells. The first signal comes from the recognition of their cognate antigen through the B-cell receptor (BCR). The second signal depends on the engagement of activating and inhibitory co-receptors that, when triggered during an immune reaction, induces a positive or a negative cell signaling pathway. The integration of these opposite signals determines the extent of B-cell activation, which determines the fate of B cells [90]. PD-1, which is involved in the negative regulation of T-cell activation and are also expressed on B cells [6,91,92]. The B cells express PD-1 upon activation and this expression is maintained on some B cells [93,94], but its importance in the process of B-cell activation/antibody production and dysfunction remains elusive.

Generalized B-cell dysfunction is an important feature of HIV-1 and pathogenic SIV infection. B cell abnormalities in HIV disease include imbalances in B-cell subsets and recent data suggest that these abnormalities are due to presence of memory B cell subsets in HIV infected that are totally absent in healthy individuals [95]. There are at least six B-cell subsets that can be clearly delineated in the peripheral blood of healthy and HIV-infected individuals [95] and nine B cells subsets according to the most common lineage/differentiation makers and relative tissue distribution [96]. Several B cell subsets are reportedly altered in HIV-infected individuals. One of the altered subsets is naive B cells [97,98], which display a decreased frequency in HIV-viremic individuals [99,100]. High levels of activated memory B cells prior to infection directly correlate with increased depletion of these cells following SIV infection in rhesus macaques as well as HIV-infected individuals [98]. These data suggest that the levels of activated memory B cells prior to infection may influence disease progression and the individuals with relatively high levels of activated memory B cells prior to HIV infection may be at higher risk for faster disease progression [101,102]. Multiple factors, including the incidence of non-HIV infections, could influence the frequency of activated memory B cells in humans and macaques. Indeed, studies in humans reported a faster rate of HIV disease progression in developing countries compared with developed countries [98,103]. Furthermore, concurrent infections with other pathogens of bacterial, helminthic, or protozoan origin have been shown to markedly affect the severity and course of disease progression [98]. The mechanisms for B-cell depletion during HIV/SIV infection are not completely understood. PD-1 has been recently emerged as a pathway involved in both HIV and SIV pathogenesis that influence B cell exhaustion. An association between loss of total memory B cells and loss of total CD4^+^ T cells was previously shown in HIV-infected individuals [97], which indicate that total memory B-cell loss may be a useful marker of disease progression. PD-1 blockade induced enhancement of memory B-cell responses in chronic SIV infection. However, in contrast to humans, PD-1 is expressed at high levels on the memory B cells of chronic SIV-infected rhesus macaques, while PD-1 levels are much lower on the memory B cells in HIV infected humans. While comparable B-cell dysfunction has been identified in SIV-infected macaques [10], the kinetics, the specific B-cell subsets that are affected, the mechanisms involved, and the role of B-cell defects in the rapid disease progression are not yet well understood. Activated memory B cells are rapidly depleted and the depletion strongly influences disease progression during chronic HIV or SIV infection [10,96]. Importantly, loss of activated memory B cells in rapid progressors was associated with a significantly higher rate of acquisition of various opportunistic infections (OIs) in SIV-infected rhesus macaques [10]. Data from SIV studies suggest that the PD-1 pathway plays an important role in the depletion and survival of activated memory B cells. These observations highlight the rapid and profound loss of activated memory B cells as an important mechanism of rapid disease progression and consequent impaired humoral immune response against SIV and non-SIV infections. Importantly, there was a strong positive correlation between the depletion of total activated memory B cells with the higher set-point viremia, which directly corresponds to the rapid disease progression [10,104]. Given that strong SIV-specific antibody responses are not detectable until several weeks post-infection [105] presumably, the activated memory B cells being depleted during the first few weeks would be primarily non–SIV-specific and constitute an integral part of pre-existing humoral immunity to non-SIV infections. The mechanisms whereby these non–SIV-specific activated memory B cells influence set-point viremia and disease progression are not completely understood. Conclusively, these results suggest that the early rapid loss of activated memory B cells in rapid disease progressors contributes to a substantially higher rate of acquisition of other OIs, which in turn contributes to hyper-immune activation and thus higher set-point viremia and rapid disease progression. Depletion of activated memory B cells from the blood likely occurs via a combination of mechanisms, including migration to other sites/tissues, decreased survival, and/or increased propensity to cell death or apoptosis. A recent study suggested that the decrease in circulating memory B cells following SIV infection mainly resulted from trafficking to lymphoid organs, with a rebound occurring once the cells returned back to the circulation from the tissues [106]. This may be the case in the slow progressors of HIV-infected humans and SIV-infected typical progressors, in which they observed a rebound in memory B cells during the chronic SIV infection, but not in rapid progressors, in which memory B cell loss was sustained [10]. Similar to humans a subset of memory B cells lack both CD27 and CD21 receptor expression in macaques [10]. In humans this subset resembles recently described tissue-like memory B cell subset in HIV-infected individuals [98]. Comparable human unconventional/tissue-like memory B cells have gained recent attention because of their expansion and accumulation in viremic HIV-infected individuals as well as their association with other OIs [97,98]. This is an intriguing subset that may have important consequences for disease pathogenesis and further studies are needed to intensely investigate these B-cell subsets. Collectively, these results strongly suggest decreased survival and increased apoptosis as a mechanism of depletion of activated memory B cells and furthermore demonstrate a role for PD-1 in B cell impairment. HIV viremia leads to B-cell exhaustion, as manifested by increased expression of multiple inhibitory receptors, altered expression of homing receptors, decreased cell division and somatic hypermutation *in vivo*, decreased proliferative and effector properties *in vitro* and enrichment of HIV-specific responses in the exhausted B-cell compartment. Although most B-cell perturbations in HIV-infected individuals are attributed to viremia and are reversible by ART, one important exception is the loss of memory B cells.

All stages of HIV infection are associated with a decrease in both the frequency of resting memory B cells and the magnitude of B-cell responses against T-cell-dependent and T-cell-independent antigens. Many B-cell perturbations observed in HIV infection also arise in various infectious and non-infectious disease settings that involve immune dysfunction. Importantly, *in vitro* stimulation of memory B cells with PD-L1 induced their deletion through apoptosis, and blockade of PD-1 pathway *in vitro* increased their survival and proliferation [10,73,107]. Furthermore, in vivo blockade of PD-1 led to increased titers of antibody to SIV antigens and other OIs [10] (Figure 2). Interestingly, Fas-mediated apoptosis of activated memory B cells was also decreased following PD-1 blockade, which suggests that both Fas-mediated and PD-1-mediated pathways of apoptosis may be interlinked. This presents the interesting possibility that Fas-mediated apoptosis could be modulated through PD-1 blockade. Given that attenuated disease course was observed in SIV-infected animals treated with a FasL-blocking antibody [10], PD-1 blockade may thus be a means to enhance humoral immune responses through modulation of both the PD-1 and Fas pathways. Given the uniform high expression of PD-1 on the majority of activated memory B cells, the possibility of PD-1 ligand expression being a determining factor in their deletion through PD-1 pathway cannot be excluded. Among the B-cell subsets, the activated memory B cells express highest level of PD-1. PD-1 expression on activated memory B cells may be related to their differentiation stage and activation status, and the majority of Ag-experienced committed B cells that may require tighter regulation has the highest PD-1 expression. This would be an important mechanism for preventing hyperactivation and autoimmunity. Indeed, studies on the effects of PD-1 signaling in B cells indicate that PD-1 may down-modulate excessive and prolonged activation by elevating the threshold for restimulation [108]. However, one can argue that pathogenic SIV infection results in accumulation of PD-1 negative memory B cells, which may also contribute to hyper-gammaglobulinemia and autoimmunity. Studies in murine model also support this therapy that PD-1 knockout mice develop autoimmune disease [109]. Hence, this may be an important mechanism to determine the development of autoimmune conditions in HIV/SIV infection.

### PD-1 ligands and immune dysfunction in HIV infection

PD-1 has two ligands, PD-L1 and PD-L2, which are members of the B7 family. PD-L1 is upregulated on macrophages and APCs in response to LPS and GM-CSF treatment, and on T cells and B cells upon TCR and B cell receptor signaling [110]. PD-L1 mRNA can be detected in various tissues such as heart, lung, thymus, spleen, and kidney during the resting stage in murine model and also in all tumor models [8,30]. PD-L2 expression is more restricted and is expressed mainly by DCs and a few tumor lines [30]. Its clear that PD-1 and its ligands negatively regulate immune responses and experiments in mouse models clearly demonstrate the key role-played by PD-1 ligands in protection from autoimmunity and excessive inflammatory responses. PD-L1 knockout mice infected with a chronic LCMV strain die from extensive immunopathology, while wild-type mice become chronically infected but survive [14]. In chronic LCMV-infected mice, virus-infected splenocytes express high levels of PD-L1, suggesting a role in ineffective CTL responses [14] and the up-regulation of PD-L1 in lymphoid organs contributes to viral persistence [14]. It is also been shown that the up-regulation of PD-L1 can attenuate pathogen-specific immune responses, such as in *Schistosoma mansoni* infection [111].

Hyper-immune activation is the hallmark of HIV and SIV infection [112,113]. During chronic HIV infection, the PD-1/PDL pathways are upregulated in order to reduce the inflammation. Consistent with this, HIV accessory proteins could also induce the expression of PD-L1 and PD-L2 on macrophages and DCs [87,114,115]. As defined earlier, PD-L1 is up-regulated in various immune cells and demonstrated a role of PD-L1 in progressive immune dysfunction. PD-L1 expression was found to be significantly elevated on DCs, monocytes and B cells in HIV-infected individuals compared with HIV-negative controls [79]. PD-L1 levels correlated with markers of disease progression, directly with viral load and inversely with CD4^+^ counts [79,85]. Exposure of myeloid DC and monocytes to HIV *in vitro* resulted in up-regulation of PD-L1 [82,86]. PD-L1 was also found to be up-regulated on myeloid DCs and monocytes in HIV-infected subjects with progressive infection, but expressed at lower levels in ART treated subjects and controllers and long-term non-progressors, HIV-encoded TLR ligands up-regulated PD-L1 on DC and monocytes [82,87] and exposure of monocytes to HIV *in vitro* resulted in PD-L1 up-regulation by an IFN-dependent mechanism [86]. Taken together, these results suggest that both viral factors and inflammatory cytokines may lead to the induction of PD-L1 on APCs, which could contribute to the functional impairment of PD-1 expressing HIV-specific CTL. Furthermore, PD-L1 can act through different receptor on immune cells, since it has been shown that PD-L1 binds to PD-1 as well as CD80 expressing cells [116] and therefore PD-L1-mediated modulation of APCs and T cells may also have a significant impact in HIV infection. More recently, it has been shown that the neutrophils in blood of HIV-1-infected individuals express higher levels of the PD-1 ligand (PD-L1) and the level of neutrophil PD-L1 expression correlates with functional impairment of HIV-specific CD8^+^ T cells and CD4^+^ T cells with higher immune senescence [88]. Furthermore, similar to the monocytes and DCs, the PD-L1 expression on neutrophils is not only induced by HIV, but also by various stimuli that are associated with HIV infection such as immune activation (LPS and IFN-α) [117]. More importantly the expression of PD-L1 by neutrophils contributes to suppression of T-cell functions by neutrophils. Taken together, these findings are consistent with a hypothesis that HIV-1 infection and ongoing microbial translocation and its products (LPS and IFN-α) induce neutrophils with an immunosuppressive activity that significantly contributes to suppression of T-cell functions in HIV-1 infection. This novel mechanism of immune suppression mediated by neutrophils may increase our understanding of HIV-1 pathogenesis and may result in designing novel therapeutics targeting the loss of immune functions in HIV-1-infected individuals. Hence, future *in vivo* studies utilizing murine and/or simian models will be critical to delineate the significance of different innate cell -mediated suppression of T-cell functions via the PD-L1/PD-1 pathway. Studies of PD-1 ligand expression and function in lymphoid tissues are necessary for a better understanding of the PD-1 pathway in HIV infection. Progress in understanding the regulation of PD-1 ligand expression in different types of innate cells in different tissues may also provide new therapeutic targets in the PD-1 pathway.

### Effect of *in vivo* blockade of PD-1 during chronic SIV infection and potential beneficial effects

In a nonhuman primate model of SIV infection, PD-1 blockade results in rapid expansion of SIV-specific CD8^+^ T cells in blood as well as in rectal mucosal tissue, suggesting that PD-1 blockade is effective in the compartment where there is higher viral replication [11]. PD-1 blockade enhanced the frequency of virus-specific CD8^+^ T cells and consistent with the rapid expansion of SIV-specific CD8^+^ T cells, which increased the frequency of SIV-specific CD8^+^ cells that expressed the proliferation marker Ki-67, perforin and granzyme-B, CD28, CD127, and CCR7, indicative of functional T cells. Interestingly, the PD-1 blockade enhance the frequency of SIV-specific CD8^+^ T cells in the Mamu A*01 negative animals, demonstrating that PD-1 blockade could enhance the frequency of virus-specific CD8^+^ T cells that are restricted by non-Mamu A*01 alleles [11]. Furthermore, PD-1 blockade enhanced the functional quality of anti-viral CD8^+^ T cells (Figure 2) by producing poly-functional CD8^+^ T cells that is capable of co-producing multiple cytokines such as IFN-γ, TNF-α and IL-2 [11]. Similar results were obtained in mouse models of HIV infection during PD-L1 blockade [54].

PD-1 blockade not only increase the proliferative capacity of virus specific CD8^+^ T cells, but also enhance the proliferation of memory B cells during chronic SIV infection [11]. Interestingly, the PD-1 blockade enhanced the proliferative and survival capacity of both SIV and non-SIV-specific B cells [10,11,118]. Suggesting that the blockade of PD-1 during SIV infection may operate to control two pathogenic components linked to SIV infection. First, PD-1 blockade on T helper cells, which may lead to the recovery of T-cell exhaustion by interfering with PD-1 and indirectly with additional molecules that are linked to T-cell exhaustion. Secondly, the masking of PD-1 on B cells may result in increased survival of B cells, particularly the activated memory B cells and newly formed plasma cells. These two events may synergize and lead to higher levels of SIV-specific antibodies. A detailed analysis of the cellular components expressing PD-1 and its ligands needs to be performed in the germinal centers of SIV-infected animals to clarify the cellular components involved in PD-1 blockade. Increase in both cellular and humoral immune responses during blockade resulted in significant reduction in plasma viremia and also prolonged the survival of SIV-infected rhesus macaques [10,11,118].

### Blockade of PD-1: PD-L signaling reduces microbial translocation

**Figure 3:**
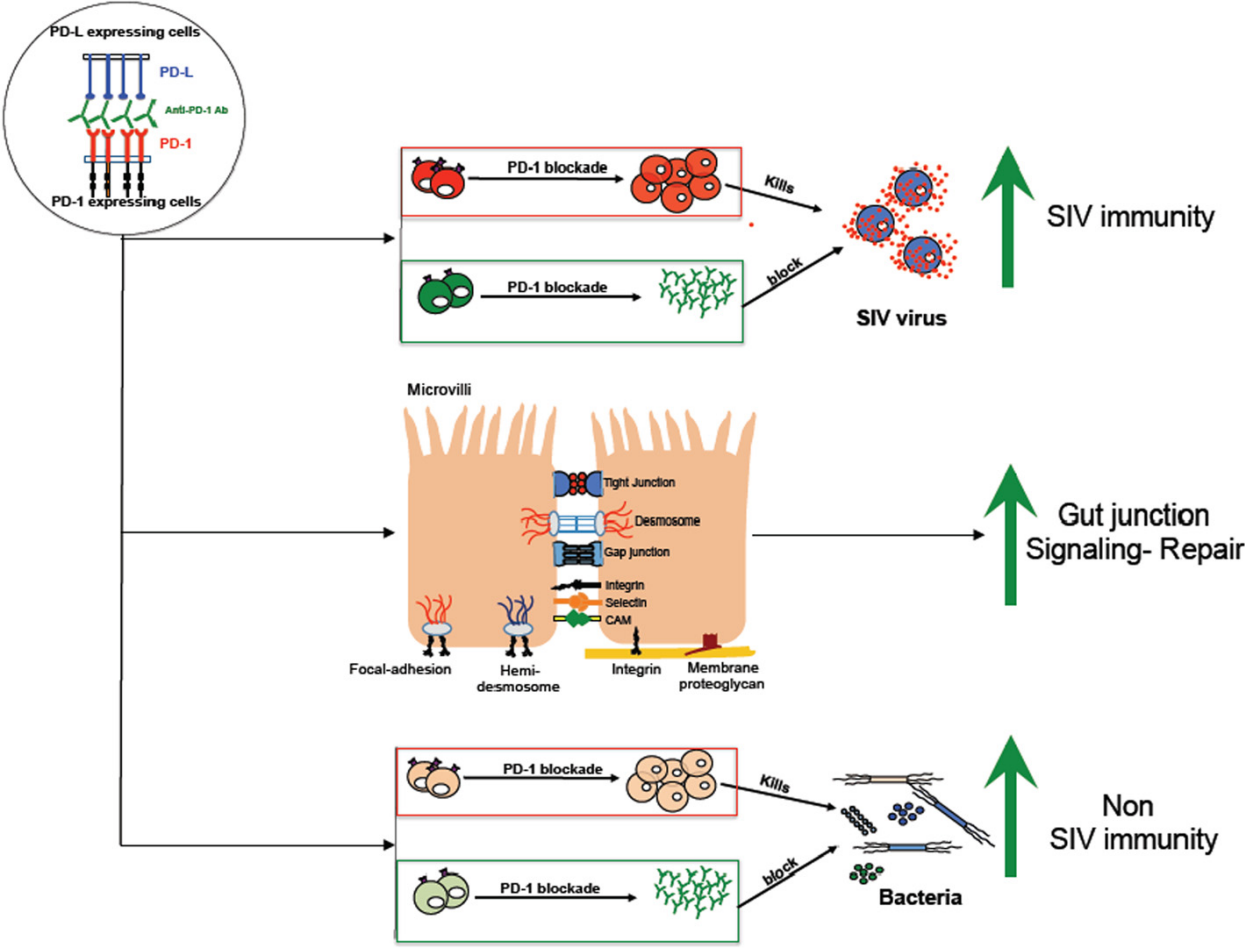
**Potential mechanisms of PD-1-directed immunotherapy.** Blockade of PD-1 pathway helps to restore T and B cell functions during chronic SIV infection. Type I IFN responses are inhibited in the blood and colorectal tissue compartments of SIV-infected Rhesus macaques following *in vivo* PD-1 blockade. Reduced type I IFN signaling was associated with enhanced expression of intestinal epithelial tight junction-associated genes and with a profound decrease in plasma LPS levels and associated immune activation, suggesting a possible gut repair and decreased pathogenic microbial translocation from gut into the blood. PD-1 blockade enhanced immunity against gut-resident pathogenic bacteria, control of gut-associated pathogenic opportunistic infections, which enhances survival of SIV-infected non-human primates.

The gastrointestinal tract is the most important target organ for HIV. Clinical presentations of acute HIV infection often include diarrhea, dehydration, chills, and loss of appetite, but just as frequently, gastrointestinal clinical manifestations are limited despite ongoing mucosal immune destruction. In advanced stages of HIV infection in humans as well as in pathogenic AIDS animal models, irreparable damage occurs to gut tissues and their barrier functions [113,119]. The major reasons underlying gut damage are massive depletion of CD4^+^ T cells that alters gut permeability and barrier functions [113]. Under normal conditions, gut microbiota is localized in the luminal surface of the gut, but following SIV/HIV infection, due to increased gut permeability, bacteria enter into the systemic circulation and migrate into various tissues of the body, as evident from increased presence of bacterial products such as lipopolysaccharides (LPS) and soluble CD14 (sCD14, possibly derived from monocytes) in blood plasma [113]. The pathogenic bacteria may non-specifically activate immune cells and cause hyper-immune activation [120]. Transcriptome as well as mRNA expression analyses of gut tissues of anti-PD-1-treated SIV-infected rhesus macaques revealed that caludin 5 (CLDN5), junction adhesion molecule 2 (JAM2), connexin 45 (CX45) and connexin 43 (CX43) junctional genes that promote intestinal integrity are highly expressed following therapy [118] (Figure 3). Hyper-immune activation promotes rapid disease progression and is partly mediated by sustained pro-inflammatory type 1 IFN-signaling in response to random microbial infection [112,121]. Suppression of infection-mediated increase in type I IFNs and IFN-stimulatory genes (ISGs) expression in non-pathogenic HIV animal models and HIV controllers as compared to the pathogenic models and HIV progressors respectively, are reportedly associated with prevention of SIV infection to advanced stages of AIDS [122]. Following PD-1 blockade, SIV-infected rhesus macaques displayed lower LPS levels in plasma besides decreased expression of ISGs in both blood and gut compartments following blockade of PD-1 [118]. Several events may underlie PD-1-mediated down-modulation of type I IFN responses. However, lower microbial burden following anti-PD-1 therapy and positive stimulation of T cells that promotes decreased sensitivity to type I IFNs and eventual lower expression of ISGs are possible mechanisms underlying lowered type I IFN responses.

### PD-1 blockade controls opportunistic microbial infections in pathogenic AIDS viral infection

PD-1 blockade during chronic SIV infection not only enhances immunity against SIV infections but also enhances both humoral and cell-mediated immune responses against various bacterial antigens [118]. Several bacterial infections commonly occur in HIV-infected patients, and *Campylobacter jejuni* represents one of the most common gram-negative motile bacteria, causing acute diarrhea in HIV-infected patients [123]. PD-1 blockade in SIV infected rhesus macaques enhanced the Campylobacter antigen-specific CD8^+^ T-cell responses and higher levels of Campylobacter-specific antibodies [10,11,118] (Figure 3). Pathogenic *Shigella spp*. (bacteria), *Trichuris trichiura* (nematode), *Candida spp.* (fungus) and *Cryptosporidum spp.* (protozoan) infections, which are commonly observed OIs during advanced stages of HIV/AIDS were decreased following inhibition of PD-1: PDL signaling [10,11,118]. Anti-PD-1-treated SIV infected rhesus macaques displayed no sign of above described OIs for three months post-termination of antibody therapy [10,11,118]. The mechanism underlying control of OIs still remains elusive and remains to be investigated whether PD-1 blockade-mediated increase in pathogen-specific protective immunity is responsible for the reported beneficial effects. Almost all animals in the anti-PD-1-treated group survived for 200 days post-therapy whereas only 45% of the animals were able to survive in the control antibody-treated group [11,118]. These suggest that combinatorial effects such as stimulation of virus-specific T and B cells, improved mucosal integrity and antimicrobial responses, reduced microbial translocation, hyper-immune activation, pro-inflammatory type I IFN responses and OIs might contribute to enhanced survival of PD-1 antibody-treated SIV-infected rhesus macaques. Conclusively, the PD-1 blockade seems to be safe and well tolerated in the macaque model of SIV infection as evident from extensive analysis of serum proteins, ions, lipids, liver and kidney enzymes, and complete blood count following the blockade. Manipulation of the immunoregulatory networks can be beneficial *in vivo* to complement ART or boost vaccine efficacy, this however warrants further investigation. Careful monitoring of potential autoimmune side effects will be critical, as demonstrated by the occurrence of an inflammatory colitis in a cancer patient treated in a recent trial with PD-1 blockade [124-126]. Interestingly, the PD-1 blockade in SIV model did not induce any significant difference in the levels of anti-nuclear antibodies (ANA) in serum following treatment with anti-PD-1 antibody. These results demonstrate that anti-PD-1 treatment during chronic SIV infection results in no observable toxicity. Studies in humans also demonstrated the safety of PD-1 blockade in patients with advanced malignancies [20,124,125]. Overall these results are highly significant in HIV infection considering the failure of blockade of a related co-inhibitory molecule CTLA-4 to expand virus-specific CD8^+^ T cells during chronic SIV infection and to reduce plasma viral load in SIV-infected macaques [127]. The therapeutic benefits of PD-1 blockade may be improved further by using combinatorial ART (cART) and/or therapeutic vaccination.

### PD-1 blockade reduces SIV viral load

The data from the non-human primate model suggest that PD-1 blockade using anti-PD-1 is well tolerated and results in rapid expansion of virus-specific CD8^+^ T cells with improved functional quality [11,19]. This enhanced T-cell immunity was seen in the blood and also in the gut mucosa, a major reservoir of SIV infection. PD-1 blockade also resulted in proliferation of memory B cells and increases in SIV envelope-specific antibody. These improved immune responses were associated with significant transient reductions in plasma viral load and also prolonged the survival of SIV-infected macaques. Interestingly, the blockade was effective during the early as well as late phases of chronic infection even under conditions of severe lymphopenia. These results demonstrate enhancement of both cellular and humoral immune responses during a pathogenic immunodeficiency virus infection by blocking a single inhibitory pathway and identify a novel therapeutic approach for control of HIV infections. However, in rhesus macaques, using a novel soluble recombinant macaque PD-1 fused to a macaque Ig-Fc (rPD-1-Fc) an alternate strategy for blocking the PD-1/PD-L1 pathway, although found effective in rescuing the effector function of SIV-specific CD4^+^ and CD8^+^ T cells during the early chronic phase of infection, has limited clinical benefit [128].

### Multiple inhibitory receptors are associated with CD8^+^ T-cell exhaustion during chronic HIV infection

A highly complex network of positive and negative signals from the co-stimulatory and co-inhibitory receptors regulates the outcome of virus-specific T-cell responses during acute and chronic viral infections [47,129,130]. Multiple inhibitory receptors are expressed in various levels on virus-specific CD8^+^ T cells (Figure 1) and play a major role in impairment of T-cell functionality during chronic viral infection [131]. It is also clear that the number and type of inhibitory receptors, which the T cells express during chronic viral infections determines the fate of functional responses to combat pathogenic infections [35,132]. Several studies have reported the importance of PD-1/PD-L pathway in functional exhaustion of CD8^+^ T cells in HIV infected individuals and SIV infected rhesus macaques [11,21,28,42,133,134]. However, only a few studies have demonstrated the simultaneous expression of several inhibitory receptors on HIV-specific CD8^+^ T cells. During chronic HIV infection, HIV-specific CD8^+^ T cells has been shown to express multiple inhibitory receptors such as PD-1, CD160, 2B4, TIM-3 and the expression of multiple inhibitory receptors correlates strongly with both HIV viral loads as well as with impaired cytokine production [29,130,135-137] (Figure 1). The increase and simultaneous expression of several co-inhibitory receptors on HIV-specific CD8^+^ T cells and the expression pattern of inhibitory receptors correlated positively with the degree of HIV-specific CD8^+^ T-cell exhaustion [29]. The biological need for the simultaneous expression of an array of inhibitory molecules on virus-specific CD8^+^ T cells still remains an enigma, since different inhibitory receptors (PD-1, CD160 and 2B4) are distinctly regulated in various T cell subsets [29,46]. Importantly, certain molecules such as 2B4 can elicit both co-stimulatory and co-inhibitory signals depending on the type of cell type expressed on. The expression level and the abundance of adaptor molecules associated with the inhibitory receptors determine the functional relevance of these molecules [138,139]. CD160 was significantly higher on naive CD8^+^ T cells from HIV infected individuals compared with cells in HIV negative individuals [46]. Unlike PD-1, CD160, TIM-3 and 2B4, the LAG-3 expression was observed only in the bulk memory CD8^+^ T cells and not elevated on HIV-specific CD8^+^ T cells demonstrating that each receptor can function independently. This functional independence could be critical *in vivo*, where inhibitory receptor expression likely varies on different cell populations and at different anatomic locations, and where the expression kinetics and distribution of their respective cognate ligands may also vary extensively [29]. Conclusively, these results from various studies raise the possibility that these co-expression patterns define signals that are necessary during CD8^+^ T-cell maturation with each receptor mediating a distinct and determinative cytosolic signal during the maturation process. The multiple inhibitory receptors were expressed to greater levels on CD8^+^ T cells from HIV infected individuals compared with those from HIV-negative individuals [29,46,47,135]. HIV-specific TCR-mediated stimulation, general immune activation, and direct functional effects of chronic antigen activation could be among the mechanisms that lead to this extensive expression pattern of multiple inhibitory receptors [32,37,42,119]. Interestingly, the suppression of HIV viral levels by anti-retroviral therapy was associated with reduced surface expression of most of these inhibitory markers on HIV-specific CD8^+^ T cells and maintenance of CD8^+^ T-cell functionality. Furthermore, the reduced expression of these markers after ART was found to be associated with undetectable viral loads, indicating that this expression pattern could also be used to predict viral suppression [29,46]. However, more studies are needed to determine whether the expression patterns of these markers can act as a surrogate to attain complete viral suppression. No correlation was found between CD4^+^ counts and simultaneous expression of multiple inhibitory receptors (PD-1, CD160, 2B4, TIM-3) either before or after ART, indicating that this pattern is not related to CD4^+^ reconstitution. Studies from humans and macaques have demonstrated a positive correlation between viral load and the PD-1 expression on HIV-specific CD8^+^ T cells. Similarly, a strong correlation was observed between plasma viral load and the simultaneous expression of multiple inhibitory receptors on virus-specific CD8^+^ T cells [29,46]. This association indicates that the simultaneous expression of these inhibitory markers could serve as a more accurate phenotypic composite to demarcate exhausted HIV-specific CD8^+^ T cells. Furthermore, the reduced expression of these markers after ART was found to be associated with undetectable viral loads, indicating that this expression pattern could also be used to predict viral suppression. More experiments, however, may be required to formally determine whether the expression patterns of these markers can act as a surrogate for attaining complete virus suppression.

Manipulations of co-stimulatory molecules were shown to alter the proliferation and survival capacity of virus-specific CD8^+^ T cells both *in vitro* and *in vivo* [14,35,140]. Several studies in mouse models have also documented simultaneous blockade of multiple inhibitory receptor interactions and revealed a synergistic effect on proliferation of virus-specific CD8^+^ T cells [129]. Similarly, several *in vitro* studies suggest that, although HIV-specific CD8^+^ T cells express several inhibitory receptors, blocking the multiple inhibitory receptors and ligand engagements reinvigorate the functional and proliferative capacity of HIV-specific CD8^+^ T cells [29]. Although studies indicate that the manipulation of multiple negative regulators of T-cell function may be more effective at restoring T-cell function than approaches targeting individual factors, but their clinical implications are yet to be elucidated. It is the question of nature versus nurture as the T cells express these negative regulators for a reason, and relieving them from the natural inhibitory effects of these molecules could have detrimental effects on the host. Experimental approaches targeting these negative regulators should first be tested thoroughly in animal models specifically looking for evidence of unrestrained immune activation or autoimmune manifestations. In addition, short-term, rather than long-term, manipulation of these negative regulators such as acute therapy to help clear infection or as adjuvants in vaccination would seem to offer a safer environment in which to test such therapy. Collectively, data from several investigators suggest that multiple co-inhibitory receptors could affect the development of HIV-specific CD8^+^ T-cell responses (Figure 1), and represent potential targets for new immune-based interventions in HIV-infected persons. These observations collectively indicate that multiple negative regulatory pathways contribute to CD8^+^ T-cell exhaustion and suggest that the pattern of inhibitory receptor co-expression is a useful correlate of the amount of T-cell exhaustion during chronic HIV infection. Thus, reversal of T-cell exhaustion could be improved by therapeutic targeting of multiple inhibitory receptor pathways and approaches to ‘fine-tune’ T-cell responses to persisting pathogens could help in combatting chronic viral infections and cancers.

## Conclusions

Chronic viral infections result in decreased function of virus-specific cellular and humoral immunity that occurs via upregulation of specific inhibitory receptors expressed on the immune cells. Several studies in mice, non-human primates, and humans have led to significant advances in our understanding of the central role of PD-1 and other negative regulatory molecules in regulating immune function, as well as the potential therapeutic benefit of blocking PD-1–PD-L interactions in enhancing CD8^+^ T cell, CD4^+^ T cell, and B cell functions. Studies in SIV macaque models using PD-1 blocking antibodies have produced promising results *in vivo* that value future continued investigation. What is more interesting in SIV model during PD-1 blockade is that, the blockade of PD-1 not only help SIV-specific immune responses, but also restore the functional quality of virus-specific CD8^+^ T cells, and also enhance the virus-specific B-cell response to non-SIV antigens. These results suggest that PD-1 blockade therapy also enhance the immune functions towards various OIs, which highlight the possibility of global restoration of various chronic antigen-specific immune responses even in conditions of massive SIV infection and severe lymphopenia. These studies have provided the basis for the development of humanized monoclonal antibodies that block PD-1 inhibitory signaling to enhance immune effector responses for the eradication of chronic infectious diseases with the potential for development of PD-1 agonists that can suppress the immune mediated pathology in autoimmune diseases. Several emerging data clearly suggest a complex pattern of co-expression of multiple inhibitory receptors on exhausted CD8^+^ T cells. More recently studies have shown synergistic effects of blockade of one or more inhibitory receptors during chronic viral infection, suggesting that combination therapy with PD-1 blockade can synergistically improve functions of exhausted CD8^+^ T cells. Determining the importance of these inhibitory pathways in diverse models and at different time points of the immune response is critical for the successful translation of potential therapies into the clinic. Additionally, although much less is known about molecular mechanisms of PD-1-mediated T-cell exhaustion, future studies elucidating additive effects of blocking PD-1 and other negative regulatory molecules and immunosuppressive cytokines will help to identify combinatorial approaches that can enhance T cell effector responses to vaccination and therapeutic interventions. Towards better understanding of exhausted T cells and developing more effective immunotherapy targeting PD-1, it is important to study how intracellular signals of PD-1 regulate T-cell exhaustion and how blocking the PD-1 pathway rejuvenates exhausted T cells. Thus therapy directed at PD-1 has significant advantage to control chronic infections such as HIV, HCV, HBV and including chronic antigens such as tumors. Future studies should help define the intracellular targets of these inhibitory pathways and test the potential of therapeutic interventions that target combinations of negative regulatory pathways during chronic viral infection.
